# Impact of an active strategy to control MRSA in a portuguese ICU

**DOI:** 10.1186/2197-425X-3-S1-A131

**Published:** 2015-10-01

**Authors:** E Molinos, G Fernandes, D Carvalho, E Gomes, R Araújo

**Affiliations:** Hospital Pedro Hispano, Medicina Intensiva, Matosinhos, Portugal

## Introduction

MRSA infection is a major health care problem. In 2008, as a component of a general strategy for infection prevention, a program to control MRSA was implemented in our ICU, consisting in pre-emptive isolation, rapid screening tests for identification of MRSA carriers and targeted decolonization with nasal mupirocin in addition to antiseptic body bathing. At the same time, screening of at-risk patients along with targeted isolation and antiseptic body washing was implemented in general wards

## Objectives

To evaluate the compliance with this strategy and the success of nasal eradication. To assess MRSA colonization prevalence and transmission rate evolution. To analyse this strategy impact on MRSA rates, oxacillin-resistance and anti-MRSA antibiotic consumption.

## Methods

We retrospectively analyzed all patients admitted to our ICU between 2010 and 2014. Data collected included demographics, nasal swab, isolation measures and nasal mupirocin prescription, staphylococcus aureus isolates and pattern of sensibility along with consumption of vancomycin, linezolid, tigecycline and daptomycin.

## Results

1753 admissions and 3149 nasal swabs were analyzed during the study period. On admission 98% (1720/1753) of patients had a nasal swab taken. We found a documented order for contact isolation in 92,5% of the positive (colonized) patients. Only 72,4% (126/174) of colonized patients started nasal mupirocin in the ICU. After treatment with nasal mupirocin 31/92 (33,7%) of patients had a negative nasal swab, and this eradication rate improved from 31,3% in 2010 to 63,6% in 2014. Prevalence of MRSA colonization on admission was 8,4%, 11,7%, 5,3%, 3,6% and 5,1% respectively from 2010 to 2014. Transmission rate was 5,2 new cases/1000 patient-days in 2010 and 5,1 in 2014. MRSA incidence was 7.39 isolates/1000 patient-days in 2011, 2.72 in 2012, 3.91 in 2013 and 2.32 in 2014. The percentage of oxacillin-resistance decreased from 59.3% in 2011 to 18.2 in 2014. Anti-MRSA antibiotic consumption was 1547 DDD/1000 patients in 2011, 1330 in 2012, 649 in 2013 and 815 in 2014

## Conclusions

MRSA incidence and methicillin resistance is decreasing in our ICU and this may be related to our strategy to control MRSA along with a multidirectional infection prevention approach. The declining in consumption of anti-MRSA antibiotics may reflect not only a reduction in clinical infections but also less MRSA coverage in the empirical treatment of nosocomial infections. The compliance with the strategy has improved but the nasal eradication rate remains low. Transmission rate of MRSA remains higher than expected, signaling that more efforts must be done in enhancing transversal infection prevention measures.
